# Healthcare services in the intersectoral coping of domestic violence against children: a scoping review

**DOI:** 10.1590/0034-7167-2025-0147

**Published:** 2025-11-21

**Authors:** Rosemeire Natsuko Shoji, Emiko Yoshikawa Egry, Karen Namie Sakata So

**Affiliations:** I Universidade de São Paulo. São Paulo, São Paulo, Brazil

**Keywords:** Child Abuse, Intersectoral Collaboration, Health Services, Preventive Program, Domestic Violence., Maltrato a los Niños, Colaboración Intersectorial, Servicios de Salud, Programa Preventivo, Violencia Doméstica.

## Abstract

**Objectives:**

to map and analyze the limits and potential of healthcare services in addressing domestic violence against children in the context of the intersectoral network in light of Sustainable Development Goal (SDG) targets.

**Methods:**

a scoping review according to the JBI method, with a search in 11 databases with publications between 2000 and 2023. Content analysis followed Bardin’s framework.

**Results:**

thirty-four studies were analyzed, predominantly quantitative (67.65%). Scientific production was small and irregular, with an average of 1.42 publications per year. The United States of America led (29.41%), followed by Brazil and the Netherlands (14.71%). Six empirical categories were identified and related to the SDG16 targets.

**Final Considerations:**

strategies such as home visits and professional training have potential, but still lack systematization. Alignment with SDG16 targets requires investments in intersectoral coordination and public policies based on the promotion of children’s rights.

## RESUMEN

**Objetivos:**

mapear y analizar los límites y potencialidades de los servicios de salud en el enfrentamiento de la violencia doméstica contra la niñez en el contexto de la red intersectorial a la luz de las metas de los Objetivos de Desarrollo Sostenible (ODS).

**Métodos:**

revisión de alcance según el método del JBI, buscando en 11 bases de datos con publicaciones entre 2000 y 2023. El análisis de contenido siguió el marco de Bardin.

**Resultados:**

se analizaron 34 estudios, predominantemente cuantitativos (67,65%). La producción científica fue pequeña e irregular, con un promedio de 1,42 publicaciones al año. Estados Unidos lideró (29,41%), seguido de Brasil y los Países Bajos (14,71%). Se identificaron seis categorías empíricas relacionadas con las metas del ODS 16.

**Consideraciones Finales:**

estrategias como las visitas domiciliarias y la capacitación profesional tienen potencial, pero aún carecen de sistematización. La alineación con las metas del ODS 16 requiere inversiones en coordinación intersectorial y políticas públicas basadas en la promoción de los derechos de la niñez.

## INTRODUCTION

**Figure 1:**
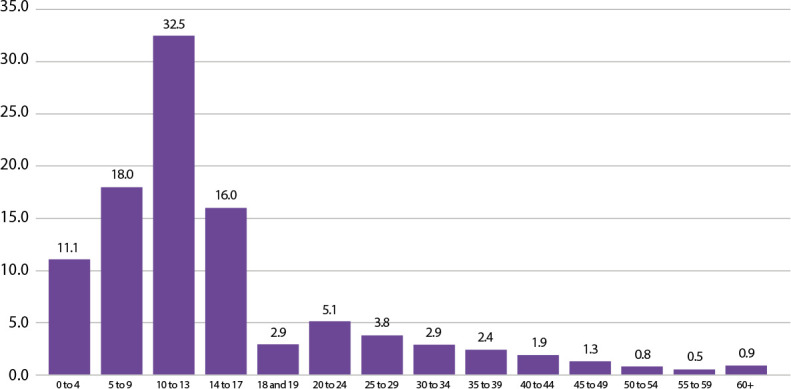
Age range of victims of rape and rape of vulnerable people (%), Brazil, 2023

*Source: Brazilian Public Security Forum, 2024.*

Domestic violence against children is a serious public health concern in Brazil and worldwide, generating high social, economic and emotional costs, in addition to perpetuating intergenerational cycles of vulnerability. Although the country has robust legislation to protect children, such as the Child and Adolescent Statute and complementary laws, structural obstacles persist in the performance of healthcare services, such as fragmentation between sectors, underreporting of cases and the absence of well-defined flows for referring victims^(1,2)^. Statistics reveal a significant increase in cases of abuse, especially sexual abuse, against girls up to 13 years of age (Figure 1), mostly perpetrated by family members, indicating the urgent need to strengthen intersectoral coordination and train professionals for early detection and humane care. Therefore, the majority of rape victims in Brazil are girls, not women^(3,4)^.

Child abuse is a multifaceted phenomenon, rooted in historical, social and cultural factors that are reproduced in families’ and institutions’ daily lives. It involves different forms of aggression - physical, sexual, psychological, and neglect - and constitutes a serious violation of children’s and adolescents’ rights, internationally recognized as a risk factor for various physical and mental health problems^(5)^. Perpetrators are, in most cases, people close to them, such as parents and guardians^(3)^, which makes it difficult to recognize the violence and break the cycle of abuse^(2)^. Children with disabilities face even greater risks, since the lack of specific data on this group makes it difficult to formulate effective interventions and implement policies aimed at their protection^(6)^.

According to data from the 18^th^ Brazilian Public Security Yearbook (2024), in 2023, more than 56,000 rapes of vulnerable people were recorded in Brazil, with 88.7% of victims being female and 61.4% up to 13 years of age. Moreover, 64.4% of perpetrators were part of children’s families. These data reinforce that the home, idealized as a space of safety and affection, can become the main place of violation and suffering for thousands of children^(4)^.

The consequences of violence in childhood are multiple and long-lasting, directly affecting neuropsychomotor development, learning, behavior and the formation of healthy bonds. Children exposed to violence are more likely to develop mental disorders, such as depression and anxiety, as well as to have academic difficulties, early use of psychoactive substances and repetition of patterns of violence in adulthood^(7,8)^.

The COVID-19 pandemic has worsened the phenomenon of violence against children by temporarily closing schools and welfare services, making it difficult to identify violence early and increasing children’s exposure to risky situations. The literature shows that social isolation, parental stress and economic overload acted as triggers for the increase in domestic violence during the pandemic. The retraction of public policies and reduction of in-person care further compromised the intersectoral response^(9,10)^.

Scientific production on the subject, although growing, is still insufficient given the magnitude of the problem. There is a lack of evaluative studies and investigations with methodological rigor that allow measuring the impact of health actions on indicators of violence against children^(2)^. Furthermore, studies point to the need for approaches that consider the intersectionality of subjects assisted - gender, race, disability, territory - as central elements for the construction of more equitable practices^(6)^.

Although healthcare services are a gateway for cases of violence, institutional response is still insufficient given the complexity of the demands. Studies point to the low resolution of teams, difficulties in identifying non-physical signs of violence, absence of defined referral flows and poor coordination with other sectors of the protection network^(1,11)^. Mandatory notification, provided for by law as an essential instrument of surveillance and protection, is still little used in daily practice by professionals who are often unaware of its purpose or fear reprisals^(12)^.

Effectively addressing violence against children requires coordinated action between the health, education, social welfare and justice sectors, as recommended in Brazilian public policies and in the international commitments undertaken by the country. The 2030 Agenda for Sustainable Development, of which Brazil is a signatory, highlights the eradication of all forms of violence against children as an ethical and political imperative for fair and sustainable development. In particular, Sustainable Development Goal (SDG) 16 proposes to “promote peaceful and inclusive societies for sustainable development, provide access to justice for all and build effective, accountable and inclusive institutions at all levels”^(13)^.

For healthcare services to contribute to achieving SDG 16, it is necessary to consolidate evidence-based practices, strengthen community ties and implement institutional protocols that guide reception, qualified listening, notification and referral of cases^(12,14)^. Human rights training, a victim-centered approach and interprofessional work are essential components for action committed to children’s dignity and well-being^(13,15)^.

Nursing, due to its reach and direct contact with the population, plays a fundamental role in the early identification of violence, in supporting victims and in monitoring cases. Studies show that trained nursing professionals can detect subtle signs of child suffering and act proactively in mobilizing the protection network. However, for this action to be effective, it is essential to guarantee institutional support, adequate consultation time and safe environments for providing care^(16,17)^.

Considering the above, it is clear that domestic violence against children is a highly relevant problem, the approach to which requires integrated responses based on scientific evidence and guided by human rights. Healthcare services must be prepared not only to address the consequences of violence, but also to act in a preventive, educational and coordinated manner. This study is justified by the need to systematize existing knowledge on the subject, identify gaps in health actions and propose ways to strengthen comprehensive protection for children. Thus, the research seeks to answer the following question: what does the literature present about the limits and potential of healthcare services to address domestic violence against children in the context of the intersectoral network?

## OBJECTIVES

To map and analyze studies on the limits and potential of healthcare services to address domestic violence against children in the context of the intersectoral network in light of SDG 16 targets, which proposes the strengthening of effective, inclusive and accountable institutions for the promotion of peace, justice and child protection.

## METHODS

**Figure 2:**
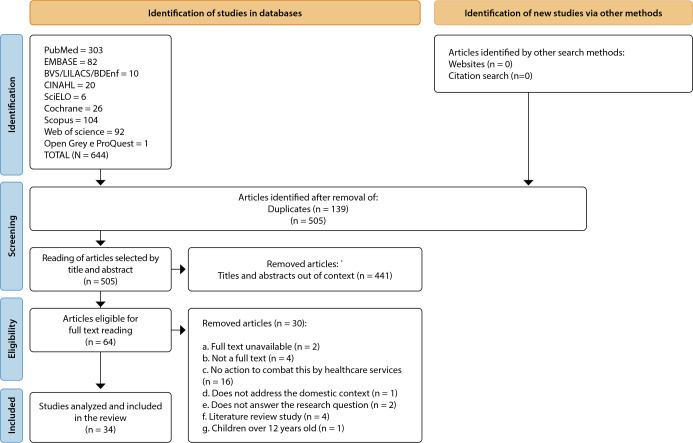
Preferred Reporting Items for Systematic Reviews and Meta-Analyses Extension for Scoping Reviews (PRISMA-ScR), 2024

This is a scoping review following the Preferred Reporting Items for Systematic Reviews and Meta-Analyses Extension for Scoping Reviews (Figure 2), whose objectives, inclusion criteria and methods were previously defined, in addition to protocol registration^(18)^. The review was prepared using the method recommended by JBI^(19)^, consisting of exploratory review^(20)^. Data selection and extraction procedures were performed from studies published in scientific databases, followed by a comparative analysis between the empirical data collected and the proposed model. The searches were carried out from July to November 2024, in journals indexed in the databases through the Coordination for the Improvement of Higher Education Personnel (In Portuguese, *Coordenação de Aperfeiçoamento de Pessoal de Nível Superior* - CAPES) portal and the Virtual Health Library (VHL), such as the Scientific Electronic Library Online (SciELO), the National Library of Medicine (PubMed) portal and EMBASE, initially as a first search attempt. This initial strategy allowed the formulation of a structured search method that was later adjusted and adapted to other databases, such as the Nursing Database (In Portuguese, *Base de Dados de Enfermagem* - BDENF), CINAHL, Cochrane, LILACS, Scopus, Web of Science, Open Grey and ProQuest.

Studies in Portuguese, English and Spanish, published since 2000, peer-reviewed and aligned with SDG 16, involving situations of violence against children in the domestic context, coping with domestic violence against children by healthcare services in the context of the intersectoral network and practices carried out by healthcare professionals linked to the intersectoral network of specialized and non-specialized care, were included. Studies on coping with violence against children in situations of war, armed conflict or in institutional shelters, descriptions of projects, protocols and programs to tackle violence against children, as well as review articles, theses, dissertations, books, organizational guidelines, letters, editorials, abstracts, institutional policies and studies with only abstracts or partial results or that were not available online, were excluded.

The analysis of the findings was conducted according to Bardin’s^(21)^ content analysis methodological framework, which is characterized by a systematic approach, although it admits a certain degree of subjectivity, using induction and intuition as strategies to deepen the understanding of the phenomena investigated. In the context of qualitative research, Bardin^(21)^ proposes that the analysis be structured in three successive stages: pre-analysis; material exploration; and treatment of results, which involves inferences and interpretations.

In this process, the theory emerges from the analysis itself, with categories progressively developed as the data are refined. According to Bardin^(21)^, categorization occurs through the classification of elements based on differentiation criteria, followed by data regrouping according to the topics that emerge from the analysis, which characterizes the definition of categories *a posteriori* ^(22)^.

This article, derived from a master’s dissertation, focuses specifically on elements related to the first and last thematic axis, with the aim of providing support for the central discussion of the study.

## RESULTS

A total of 34 documents were included (Figure 2), predominantly with a quantitative approach (67.65%). Table 1 presents the distribution of articles published by country of origin, including the absolute number of publications, the corresponding percentage and the overall total. The set includes 34 articles distributed among 13 countries, demonstrating a considerable breadth in the origin of the research. The studies revealed the predominance of international publications with a concentration of productions in the United States of America (USA) and a low density of Latin American research.

**Table 1:** Summary of included articles

Country of origin	N.º	%
United States of America	10	29.41
Brazil	5	14.71
Netherlands	5	14.71
United Kingdom	4	11.76
New Zealand	2	5.88
Germany	1	2.94
Western Australia	1	2.94
Canada	1	2.94
Spain	1	2.94
Japan	1	2.94
Paraguay	1	2.94
Sweden	1	2.94
Vietnam	1	2.94
**Total**	**34**	**100**

The USA leads with ten studies, representing 29.41% of the total. Brazil and the Netherlands are tied for second place, each with five studies, corresponding to 14.71% each. The United Kingdom is in third place, with four studies (11.76%), followed by New Zealand, with two studies (5.88%). Countries that present one study each (2.94%) are Germany, Western Australia, Canada, Spain, Japan, Paraguay, Sweden and Vietnam.

**Figure 3:**
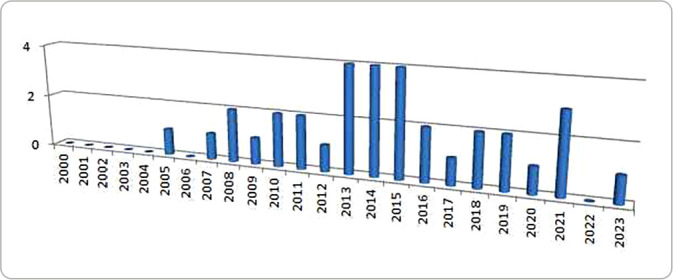
Articles selected in the scoping review according to the year of publication

Figure 3 shows an irregular and insufficient scientific production given the severity and global scope of domestic violence against children, especially in countries with a high prevalence of the problem, such as Brazil. A small number of publications can be observed, with an average of 1.42 publications per year (2000-2023), which limits the construction of contextually appropriate evidence to support more effective public policies and care practices in international and national scenarios. This gap highlights the need to promote interdisciplinary and intersectoral research in the field of health, with a focus on children’s rights and aligned with SDG 16 targets.

**Figure 4:**
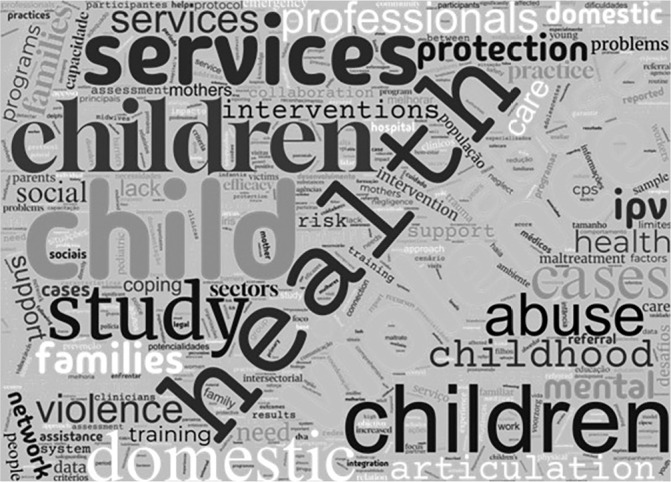
Word cloud of selected studies

The research data are available in Chart 1, which presents a summary of included studies, covering characteristics such as year of publication, country of origin, title, authors, objectives and main results. Additionally, the most frequently encountered words in the included studies are represented in Figure 4 by the word cloud.

**Chart 1:** Summary of articles included in the review

DOC	YEAR COUNTRY	TITLE	AUTHORS	OBJECTIVE	MAIN RESULTS
D01	2005 United States of America	Behaviors of children who are exposed and not exposed to intimate partner violence: an analysis of 330 black, white, and Hispanic children^(23)^	Judith M. McFarlane, Janet Y. Groff, Jennifer A. O’Brien & Kathy Watson	Determine whether a treatment program offered to abused mothers positively affects their children’s behavior.	Children exposed to domestic violence improved their behavior after 24 months, especially between 18 months and 5 years. Assessments and referrals were effective, but adolescents still experienced high levels of depression and anxiety. Most exceeded baseline standards after 24 months.
D02	2007 United States of America	What criteria do Child Protective Services investigators use to substantiate exposure to domestic violence?^(24)^	Carol Coohey	Determine whether child protective services investigators apply a recognizable set of criteria to demonstrate that perpetrators and victims of assault expose their children to domestic violence.	The study used consistent criteria to identify cases of domestic violence, prioritized holding perpetrators accountable and determined that children should only be removed in situations of imminent risk, focusing on circumstances that pose the greatest threat to their safety.
D03	2008 Brazil	*Detecção de maus-tratos contra a criança: oportunidades perdidas em serviços de emergência na cidade do RJ, Brasil* ^(25)^	Anna Tereza Miranda Soares de Moura, Claudia Leite Moraes, Michael Eduardo Reichenheim	Estimate the magnitude of psychological violence, physical violence and neglect experienced by children attending these services.	Active search revealed high underreporting, requiring better training, organization of services and integration to protect child victims of violence.
D04	2008 New Zealand	Improving detection and quality of assessment of child abuse and partner abuse is achievable with a formal organisational change approach^(26)^	Russell Wills, Miranda Ritchie, Mollie Wilson	Improve the detection and quality of assessment of child and partner abuse in a healthcare service.	The study found a significant increase in reporting and identification of child abuse by healthcare services, as well as improvements in clinical records and referrals. The training of more than 700 professionals resulted in increased confidence in screening and interventions, demonstrating sustainability and continued growth in practices.
D05	2009 Paraguay	Child Abuse: the Experience of the Multi-Disciplinary Mental Health Unit of the Niños de Acosta Ñú General Pediatric Hospital^(27)^	Lourdes Zelaya de Migliorisi, Lic. Estela González, Lic. Lina Piris de Almirón	Describe the epidemiological characteristics, the most frequent reasons for consultation and the diagnoses associated with child abuse.	Domestic violence and depressive symptoms were the main reasons for child consultations, with 56.5% of victims between 4 and 14 years old. Pediatricians need training for a multidisciplinary approach, since 99% of children knew the perpetrator, and neglect was the most frequent type of mistreatment.
D06	2010 United States of America	Children who witness violence: what services do they need to heal? ^(28)^	Danny Willis, Joellen W. Hawkins, Carole W. Pearce, Jaime Phalen, Meredith Keet, Cristen Singer	Obtain information from focus groups about interventions that participants with personal and professional experience with children who witness violence believe may be helpful for children, adolescents and their families.	Multisectoral interventions were important to promote resilience in children exposed to violence, emphasizing professional training, emotional support with creative programs, and collaborative community coalitions to effectively address violence.
D07	2010 United Kingdom	Safeguarding children and public health: midwives’ responsibilities^(29)^	Anne Lazenbatt	Compare and contrast how midwives working in hospital or community settings are currently responding to the co-occurrence of domestic and child abuse.	The research highlighted the strong link between domestic violence and child abuse, highlighting the high incidence of assault during pregnancy and the heightened risks for young children. It also identified gaps in midwives’ training and reporting capacity, highlighting the urgent need for capacity building and multidisciplinary approaches for a more effective response.
D08	2011 Netherlands	Addressing risk factors for child abuse among high risk pregnant women: design of a randomised controlled trial of the nurse family partnership in Dutch preventive health care^(30)^	Jamila Mejdoubi, Silvia van den Heijkant, Elle Struijf, Frank van Leerdam, Remy HiraSing, Alfons Crijnen	Assess whether VoorZorg is as effective in the Netherlands as it is in the United States of America.	The VoorZorg program has been shown to be effective in preventing child abuse and domestic violence by providing ongoing support and structured interventions to mothers, addressing risk factors through home visits and parenting skills training. It has improved maternal and child health, created a safer environment, and reduced the perpetuation of cycles of violence.
D09	2011 Spain	Maternal experiences of childhood abuse and intimate partner violence: Psychopathology and functional impairment in clinical children and adolescents^(31)^	Jenniffer K. Miranda, Nuria de la Osa, Roser Granero, Lourdes Ezpeleta	Examine the independent effects of maternal child abuse and intimate partner violence on psychopathology and functional impairment in children; assess the potential moderating and mediating roles of individual and family factors in these relationships; explore the potential cumulative effects of maternal child abuse and intimate partner violence on child outcomes.	Children exposed to domestic violence, including maternal abuse and physical punishment, are at high risk of psychological disorders, such as behavioral problems and internalizing behavior. Cumulative exposure to multiple forms of violence exacerbates these impacts, highlighting the need for early interventions and support for mothers to mitigate the negative effects.
D10	2012 Germany	Key role in the prevention of child neglect and abuse in Germany: continuous care by qualified family midwives^(32)^	Gertrud M. Ayerle, Katja Makowsky, Beate A. Schucking	Gain in-depth knowledge of psychosocial and health-related vulnerable families and the “portfolio” of care that family midwives provide.	Domestic violence is a significant risk factor faced by many families served, with 21.5% reporting experiences of violence and complex patterns of vulnerability. Collaboration with youth welfare services and ongoing educational activities were essential to address these challenges and improve maternal and child care.
D11	2013 United States of America	A brief intervention affects parents’ attitudes toward using less physical punishment^(33)^	Antwon Chavis, Julia Hudnut-Beumler, Margaret W. Webb, Jill A. Neely, Len Bickman, Mary S. Dietrich, Seth J. Scholer	Determine whether a brief intervention, integrated into the primary care visit, can affect parental attitudes toward using less physical punishment.	A brief multimedia intervention reduced parental attitudes favorable to the use of physical punishment and increased the use of nonviolent discipline strategies.
D12	2013 Netherlands	A new protocol for screening adults presenting with their own medical problems at the Emergency Department to identify children at high risk for maltreatment^(34)^	Hester M. Diderich, Minne Fekkes, Paul H. Verkerk, Fieke D. Pannebakker, Mariska Klein Velderman, Peggy J.G. Sorensen, Paul Baeten, Anne Marie Oudesluys-Murphy	Assess whether a triage protocol for adults presenting for care in the Emergency Department can identify children at high risk for formal treatment.	Implementation of the Hague Protocol significantly increased the detection of child abuse cases, with a high confirmation rate (91%) and revealed that most cases were previously unknown to the authorities. Detections mainly included children exposed to domestic violence, demonstrating the effectiveness of the protocol in identifying new cases of child abuse.
D13	2013 United States of America	Building healthy children: evidence-based home visitation integrated with pediatric medical homes^(35)^	Heather A Paradis, Mardy Sandler, Jody Todd Manly, Laurie Valentine	Prevent child abuse, improve the health of parents and children, and enhance family functioning.	The Building Healthy Children program reduced children’s exposure to domestic violence and the need for reporting to Child Protective Services, ensuring high participant retention (85%) and preventing new cases of abuse, in addition to promoting preventative care for healthy development.
D14	2013 Brazil	*As possibilidades de enfrentamento da violência infantil na consulta de enfermagem sistematizada* ^(36)^	Maíra Rosa Apostólico, Paula Hino, Emiko Yoshikawa Egry	Identify the limits and potential of the International Classification of Nursing Practices in Public Health^®^ in nursing consultations with children who are victims of domestic violence.	The International Classification of Nursing Practices in Public Health^®^ nomenclature presented limitations in recognizing violence, especially in cases of neglect and child abuse, making reporting and continuity of care difficult. The lack of precision in diagnoses highlighted the need for additional attributes and greater integration with protocols to ensure more effective care.
D15	2014 Netherlands	Facilitators and barriers to the successful implementation of a protocol to detect child abuse based on parental characteristics^(37)^	Hester M. Diderich, Mark Dechesne, Minne Fekkes, Paul H. Verkerk, Fieke D. Pannebakker, Mariska Klein Velderman, Peggy J.G. Sorensen, Simone E. Buitendijk, Anne Marie Oudesluys-Murphy	Explore whether the Hague Protocol guidelines can be successfully implemented and identify critical enablers or barriers to implementation.	Implementation of the Hague Protocol significantly increased referrals of child abuse cases to Reporting Center for Child Abuse and Neglect, but low confirmation rates in some regions and lack of training for professionals compromised effectiveness. The presence of an implementation coach and ongoing training of professionals were essential to improving detection and response to child abuse in a variety of contexts.
D16	2014 United States of America	Health, emergency department use, and early identification of young children exposed to trauma^(38)^	Yvonne Humenay Roberts, Cindy Y. Huang, Cindy A. Crusto, Joy S. Kaufman	Describe the prevalence of physical health symptoms and health-related problems in young children affected by trauma and to predict whether or not children who experience trauma are more likely to be affected by health-related problems.	The high prevalence of trauma in children exposed to domestic violence has led to increased use of healthcare services, highlighting the need for appropriate screening and intervention in emergency departments. Specialized training for healthcare professionals has been essential to improve early identification and care of children affected by trauma.
D17	2014 United Kingdom	Keeping the focus on children: the challenges of safeguarding children affected by domestic abuse^(39)^	Sue Peckover, Fiona Trotter	Examine the challenges faced by professionals in protecting children affected by domestic violence, particularly those working in universal and additional support services (DH 2009).	Professionals recognize the impact of domestic violence on children, but struggle to respond due to a lack of specialized services, adequate training, and a focus on children’s needs. In addition, delegating responsibility to other agencies creates gaps in care.
D18	2014 Netherlands	Support and monitoring of families after child abuse detection based on parental characteristics at the Emergency Department^(40)^	H M Diderich, F D Pannebakker, M Dechesne, S E Buitendijk, A M Oudesluys-Murphy	Investigate what happened to families three months after abuse was detected.	The use of the Hague Protocol in emergency care was effective in identifying cases of child abuse, with 91% of cases referred being confirmed. However, there were limitations in ongoing monitoring and collaboration between services, leading to frequent re-referrals and indicating the need for a centralized monitoring system to prevent recurrences.
D19	2015 United States of America	Child maltreatment and risk patterns among participants in a child abuse prevention program^(41)^	Jennifer Y. Duffy, Marcia Hughes, Andrea G. Asnes, John M. Leventhal	Examine the relationship between parental risk factors and Child Protective Service reporting status and number of reports in families in a statewide prevention program.	Families at high social risk who were served by a child abuse prevention program and reported to the Child Protection Service presented high parental risk. A history of violence and criminality of parents, the presence of multiple caregivers and at least two of the six risk factors were associated with the occurrence or recurrence of maltreatment.
D20	2015 Netherlands	Detecting child abuse based on parental characteristics: Does The Hague Protocol cause parents to avoid the Emergency Department? ^(42)^	Hester M. Diderich RN, Minne Fekkes, Mark Dechesne, Simone E. Buitendijk MD, Anne Marie Oudesluys-Murphy	Investigate whether implementation of the Hague Protocol may reduce emergency medical care.	The study revealed that, after the implementation of the Hague Protocol, the number of patients seen in the emergency department remained high and even increased, indicating that the protocol does not turn patients away.
D21	2015 United States of America	Intimate Partner Violence Programs in a Children’s Hospital: Comprehensive Assessment Utilizing a Delphi Instrument^(43)^	Kimberly A. Randell, Sarah E. Evans, Donna O’Malley, M. Denise Dowd	Conduct an initial assessment of intimate partner violence practices in a pediatric hospital system.	The study identified limitations in domestic violence screening and response practices at the hospital, with low scores on intervention practices, lack of training and standardized protocols, and institutional and individual barriers that hinder the implementation of a coordinated and effective approach.
D22	2015 Brazil	*Violência infantil: uma análise das notificações compulsórias, Brasil 2011* ^(44)^	Susana Maria Moreira Rates, Elza Machado de Melo, Márcio Dênis Medeiros Mascarenhas, Deborah Carvalho Malta	Analyze reports of violence against children aged 0 to 9 years, recorded by public healthcare services in Brazil.	The study revealed that domestic violence against children in Brazil occurs mostly in the family environment, with a high prevalence of neglect and repeated abuse, highlighting the need for intersectoral actions, professional training and interventions aimed at parents and caregivers for effective child protection.
D23	2016 New Zealand	Are mental health services getting better at responding to abuse, assault and neglect? ^(45)^	Read J, Sampson M, Critchley C	Determine whether staff responses to reports of abuse have improved since the introduction of a trauma policy and training program.	The study revealed that despite greater inclusion of abuse in treatment plans following trauma-informed policies, significant gaps remain, such as low referral rates, inadequate responses to neglect and a lack of links between child abuse and mental health problems, highlighting the need for more effective protocols.
D24	2016 Brazil	*Impacto da violência por parceiro íntimo no uso de cuidados primários de saúde para crianças: evidências do RJ, Brasil* ^(46)^	Claudia Leite de Moraes, Aline Gaudard e Silva de Oliveira, Michael Eduardo Reichenheim	Assess whether severe physical violence between intimate partners after childbirth affects the number of pediatric consultations in Primary Healthcare units during this period.	Mothers who have experienced severe intimate partner violence after childbirth are less likely to attend pediatric services, which is detrimental to child development. The impact is greater in children with health problems. Early detection of violence by healthcare professionals is crucial for protection and appropriate care.
D25	2017 United Kingdom	Training on domestic violence and child safeguarding in general practice: a mixed method evaluation of a pilot intervention^(47)^	Natalia V. Lewis, Cath Larkins, Nicky Stanley, Eszter Szilassy, William Turner, Jessica Drinkwater, Gene S. Feder	Test and assess the feasibility, acceptability and direction of change in short-term outcome measures of a pilot evidence-based training program on domestic violence and child protection for general practice staff.	Researching Education to Strengthen Primary care on Domestic Violence and Safeguarding training significantly improved clinicians’ knowledge and confidence, but had limited impact on clinical behavior change and faced challenges in cross-sector collaboration, highlighting the need for refinement and greater support for effective implementation.
D26	2018 Japan	Cumulative risk effect of household dysfunction for child maltreatment after intensive intervention of the child protection system in Japan: a longitudinal analysis^(48)^	Hirotsuna Ohashi, Ichiro Wada, Yui Yamaoka, Ryoko Nakajima-Yamaguchi, Yasukazu Ogai, Nobuaki Morita	Investigate how family dysfunction and multiple types of maltreatment predict recurrence of child maltreatment following interventions in the child protection system in Japan.	Investigate how family dysfunction and multiple types of maltreatment predict recurrence of child maltreatment following interventions in the child protection system in Japan.
D27	2018 Brazil	*Proteção de crianças e adolescentes* *vítimas de violência: a visão dos profissionais de um serviço especializado* ^(49)^	Priscila Arruda da Silva, Valéria Lerch Lunardi, Rodrigo Dalke Meucci, Simone Algeri	Understand the obstacles faced by professionals in working in a network and the challenges of their work at the Specialized Reference Center for Social Assistance in a municipality in the extreme south of Brazil.	The fragmentation of the service network, bureaucracy, lack of resources and ineffective communication compromise the protection of child victims of violence. Overworked professionals, without adequate training and with insufficient support face difficulties that reduce the effectiveness of services.
D28	2019 Western Australia	Hospitalisations for maternal assault are associated with increased risk of child protection involvement^(50)^	Carol Orr, Colleen Fisher, Scott Sims, David Preen, Rebecca Glauert, Melissa O’Donnell	Examine the risk of allegations of child abuse in children whose mothers were hospitalized due to an assault.	Children of mothers hospitalized for abuse, especially during pregnancy, were at significantly higher risk of allegations of abuse. This highlights the urgency of early interventions, intersectoral collaboration and increased awareness among professionals to support vulnerable families.
D29	2019 Sweden	Reduced psychiatric symptoms at 6 and 12 months’ follow-up of psychotherapeutic and psychoeducative group interventions for children exposed to intimate partner violence^(51)^	Karin Perneboa, Mats Fridell, Kjerstin Almqvist	Investigate the long-term outcomes of two established group interventions for children exposed to intimate partner violence and their non-abusing parents.	The study highlighted sustained improvements in child stress symptoms and behavior following domestic violence interventions, with greatest benefit for children with severe traumatic symptoms. Continued improvements were influenced by maternal psychological health and there was little re-exposure to violence.
D30	2020 Vietnam	Clinician Response to Child Abuse Presentations in the Vietnamese Hospital Emergency Setting^(52)^	Tara Flemington, Cathrine Fowler, Quang Nhat Tran, Jennifer Fraser	Explore the knowledge, practice and experience of clinicians when faced with presentations of child abuse in a pediatric emergency setting.	The study found that healthcare professionals in Vietnam face barriers such as lack of training, inadequate protocols and difficulties in documenting child abuse. Despite this, they have developed innovative methods of protection and emphasize the need for training and clear guidelines to improve response to cases.
D31	2021 United States of America	Evaluating child maltreatment and family violence risk during the COVID-19 Pandemic: Using a telehealth home visiting program as a conduit to families^(53)^	Lindsey Rose Bullinger, Stevan Marcus, Katherine Reuben, Daniel Whitaker, Shannon Self-Brown	Examine how families with young children are affected by the challenges of the COVID-19 pandemic and to assess changes in professionals’ perceptions of child maltreatment risk due to these unusual circumstances.	The study found that the COVID-19 pandemic significantly increased the risk of child maltreatment, driven by parental stress, financial hardship and lack of social distancing. The effectiveness of the SafeCare program was highlighted as an important intervention, but insufficient given the barriers faced by vulnerable families.
D32	2021 United Kingdom	‘It felt like there was always someone there for us’: Supporting children affected by domestic violence and abuse who are identified by general practice^(54)^	Jessica Roy, Emma Williamson, Katherine Pitt, Nicky Stanley, Mei-See Man, Gene Feder, Eszter Szilassy	Determine whether IRIS+ (Enhanced Identification and Referral to Improve Safety) training and advocacy support has improved the way clinicians respond to children and young people affected by domestic violence and abuse.	The study revealed significant gaps in identifying and referring children exposed to domestic violence, with barriers such as lack of clarity among professionals and fear among mothers. Despite this, specialized support brought important benefits, improving child well-being and family communication.
D33	2021 Canada	Screening for intimate partner violence in the early postpartum period: Maternal and child health and social outcomes from birth to 5-years post-delivery^(55)^	Tamara L. Taillieu, Douglas A. Brownridge, Marni Brownell	Examine maternal and child health and social outcomes from birth to 5 years postpartum associated with positive (vs. negative) maternal intimate partner violence screening around the time of delivery.	Children exposed to domestic violence face increased risks of attention deficit hyperactivity disorder, respiratory infections, hospitalizations for injuries and impaired development, while mothers experience poor mental and physical health. These findings highlight the need for integrated health and social protection interventions to support affected families.
D34	2023 United States of America	Evaluation of Children after Caregiver Intimate Partner Violence: A Qualitative Study of Barriers, Facilitators, and Traumaand Violence-Informed Care^(56)^	Gunjan Tiyyagura, Nicole Clayton, Paula Schaeffer, Marcie Gawel, John M. Leventhal, Kristen Hammel, Karen Jubanyik, Destanee Crawley, Ashley Frechette, Daniel M. Lindberg, Tami Sullivan, Andrea Asnes	Identify barriers and facilitators in the assessment of children exposed to intimate partner violence and develop a strategy to optimize assessment.	Medical assessment of children exposed to domestic violence has helped identify physical abuse and connect caregivers to support resources, despite barriers such as lack of data and parental resistance. Better collaboration between services and a trauma-informed approach can improve care and support for these families.

Six empirical categories were identified and linked to SDG 16 targets: 1. Prevention and support for domestic violence and child abuse; 2. Interventions based on professional training and multidisciplinarity; 3. Network challenges and professional barriers; 4. Risks and consequences of violence; 5. Importance of community and intersectoral approaches; and 6. Insufficient monitoring and documentation (Chart 2).

**Chart 2:** Analysis of empirical categories in light of the targets of Sustainable Development Goal 16

CATEGORIES	TARGET 16.1	TARGET 16.2	TARGET 16.3
1. Prevention and support for domestic violence and child abuse	Focus on preventive interventions and support programs that aim to reduce domestic violence and child abuse, aligning with the goal of reducing violence.	Focus on eliminating child abuse.	---
2. Interventions based on professional training and multidisciplinarity	---	Professional training and a multidisciplinary approach help to identify and combat child abuse.	Promote access to justice through qualified services.
3. Network challenges and professional barriers	Overcoming barriers requires training, intersectoral governance and integrated systems to ensure effective and humane responses to violence.	Strengthening protocols, integrating services and investing in professional training are essential actions to improve child protection.	Address obstacles to access to justice and care, directly relating to the promotion of the rule of law and equal access to justice.
4. Risks and consequences of violence	Analyze the impacts and risks of violence, contributing to the understanding needed to reduce the harm associated with this violence.	Combat child abuse.	---
5. Importance of community and intersectoral approaches	Community and intersectoral approaches are key to reducing violence.	The aforementioned cross-sectoral and community initiatives are key to creating effective safety nets, strengthening community resilience and preventing all forms of child abuse.	Ensure access to justice.
6. Insufficient monitoring and documentation	---	Proper documentation is crucial to ensuring that child abuse is reported and addressed.	Inadequate documentation and lack of reporting limit institutional response to abuse cases. Ensure effective legal processes and victim protection, contributing to strengthening the rule of law.

*Source: own authorship, 2025; GT Agenda 2030, 2024.*

These categories demonstrate that the limits faced by teams are not restricted to the individual level, but involve systemic and structural barriers (D01, D02, D03, D04, D07), such as discontinuity of policies, absence of intersectoral protocols and fragility of support networks (D10, D12, D14, D15, D17, D19, D20, D22, D23, D24, D26, D27, D33, D34) (Chart 1).

The categories indicated that potentials, such as routine screenings (D03, D04, D12, D15, D18, D21, D24, D33), home visits (D08, D10, D11, D13, D19, D31) and educational programs (D06, D07, D08, D09, D14, D17, D27, D29, D31, D33), are effective methods for early detection of cases of child abuse and domestic violence, but require highly trained professionals and significant resources (D10, D17, D19, D31, D34).

## DISCUSSION

The studies revealed a recurring pattern of limitations, such as disarticulation between sectors of the protection network, underreporting of cases, lack of standardized protocols and insufficient ongoing training of healthcare professionals^(12)^. These obstacles were recurrent in the empirical categories related to intersectoral disarticulation, underreporting of cases and the lack of systematic actions to monitor victims^(17,23)^.

The lack of coordination between sectors of the protection network compromises the flow of referrals and monitoring of cases, reflecting a fragmented and poorly effective care model^(23)^. The shortage of trained professionals, combined with limited institutional resources, contributes to the invisibility of situations of violence and revictimization of children in the very services that should be providing them^(17)^. Another critical aspect identified was the exclusion of children’s voices in the formulation of public policies, which maintains a cycle of institutional silencing of victims^(10)^.

The COVID-19 pandemic has exacerbated the vulnerability scenario by limiting children’s access to protective services and increasing the time they are exposed to perpetrators. Social isolation, parental stress, and the retraction of public policies acted as additional risk factors during the pandemic period^(6,23)^. Children from racial and ethnic minorities were disproportionately affected, although the lack of disaggregated data compromises more accurate analyses of the impact of structural inequalities^(6)^.

Despite the limitations, some initiatives stand out as potential for healthcare services. The authors identified promising experiences, such as home visits carried out by nursing professionals, educational activities in schools, humanized care in emergency services and intersectoral training programs, which contributed to strengthening the protection network. Such practices, however, are still isolated and lack institutionalization, adequate financing and systematic monitoring of results^(18)^.

The use of the International Classification of Nursing Practices in Public Health^®^ has contributed to qualifying the identification of situations of violence, through the systematization of clinical listening and coordination with social assistance services. This tool strengthens active surveillance, adequate recording of cases and the planning of more effective interventions^(24)^.

Home visiting programs carried out by specialized nurses, such as VoorZorg, have demonstrated a positive impact on violence prevention by promoting the strengthening of family ties and longitudinal monitoring of families at risk^(25)^. Positive parenting has emerged as a relevant protective factor, especially in contexts of high social vulnerability^(8)^.

From a therapeutic perspective, strategies such as art therapy have proven effective in addressing childhood trauma, allowing children to express their emotions through symbolic language, which favors the reconfiguration of violent experiences and the reconstruction of self-esteem. These interventions, although specific, demonstrate potential for incorporation into multidisciplinary care practices^(24)^.

Furthermore, the low scientific production on the subject, with uneven geographical distribution and a predominance of studies with a modest methodological level, indicates a significant gap in the field of public health and nursing research. The average of only 1.42 publications per year over 24 years, as revealed by the data in this review, is incompatible with the severity and magnitude of the problem, especially in countries like Brazil, which are among the most affected by violence against children. This scarcity reinforces the need for investment in analytical, evaluative and methodologically rigorous studies capable of supporting effective public policies^(26)^.

The findings are in direct dialogue with SDG 16 targets, which proposes the strengthening of inclusive, effective and accountable institutions. The gap between the commitments made by Brazil and institutional practice indicates the urgent need for structured public policies, with resource allocation, qualified training and collaborative governance^(13)^.

### Study limitations

The study had some limitations, such as the time frame adopted, which included studies published since 2000. This choice was based on the Statute of Children and Adolescents validity in Brazil and the adoption, at the international level, of the United Nations SDGs, which increased the visibility of child protection agendas. Another limitation was the exclusion of studies that did not clearly distinguish the child population from the adolescent population, aiming to focus exclusively on children, respecting the age groups defined in the literature and in the protection policy. Our understanding is that there are relatively significant differences in terms of care and ways of dealing with domestic violence between these two populations.

### Contributions to nursing, public health or public policies

The study detected knowledge gaps regarding the topic and subtopics addressed, especially intensive, analytical and current studies on policies and practices for intersectoral network-based confrontation of domestic violence against children.

## FINAL CONSIDERATIONS

Domestic violence against children poses urgent challenges to public health, requiring more qualified and intersectoral responses. This study reinforces the importance of repositioning healthcare services as active agents in child protection, not only as instances of clinical care, but as strategic structures for surveillance, listening and referral.

Given the magnitude of the problem, it is imperative that health actions are aligned with ethical and international commitments, such as those established in the 2030 Agenda, especially SDG 16, which demands effective, fair and inclusive institutions. The recognition of children as subjects of rights should guide the formulation of public policies and the organization of care, ensuring integrated and evidence-based responses.

## Data Availability

The research data are available in a repository: https://doi.org/10.5281/zenodo.16423368.
