# Supplementary material for: Healthcare services in the intersectoral coping of domestic violence against children: a scoping review
Source: Rev Bras Enferm. 2025 Nov 21;78(Suppl 2):e20250147. doi: 10.1590/0034-7167-2025-0147 (PMC12659915; doi:10.1590/0034-7167-2025-0147)
Supplement: Translation

# Translation: Serviços de saúde no enfrentamento intersetorial da violência doméstica infantil: revisão de escopo

**DOI:** 10.1590/0034-7167-2025-0147pt

**Authors:** Rosemeire Natsuko Shoji, Emiko Yoshikawa Egry, Karen Namie Sakata So

**Affiliations:** I Universidade de São Paulo. São Paulo, São Paulo, Brasil

**Keywords:** Maus-Tratos Infantis, Rede Intersetorial, Serviços de Saúde, Programas de Prevenção, Violência Doméstica.

## Abstract

**Objetivos:**

mapear e analisar os limites e potencialidades dos serviços de saúde no enfrentamento da violência doméstica contra crianças no contexto da rede intersetorial à luz das metas do Objetivo de Desenvolvimento Sustentável (ODS).

**Métodos:**

revisão de escopo, conforme método do JBI, com busca em 11 bases de dados com publicação entre os anos 2000 e 2023. A análise de conteúdo seguiu o referencial de Bardin.

**Resultados:**

foram analisados 34 estudos, predominantemente quantitativos (67,65%). A produção científica foi pequena e irregular, com média de 1,42 publicações anuais. Os Estados Unidos da América lideraram (29,41%), seguidos por Brasil e Holanda (14,71%). Seis categorias empíricas foram identificadas e relacionadas às metas do ODS16.

**Considerações Finais:**

estratégias como visitas domiciliares e capacitação de profissionais apresentam potencial, mas ainda carecem de sistematização. O alinhamento às metas do ODS16 requer investimentos em articulação intersetorial e de políticas públicas baseadas na promoção dos direitos da criança.

## INTRODUÇÃO

A violência doméstica contra crianças é configurada como um grave problema de saúde pública no Brasil e no mundo, gerando elevados custos sociais, econômicos e emocionais, além de perpetuar ciclos intergeracionais de vulnerabilidade. Apesar de o país possuir uma legislação robusta para proteção da infância, como o Estatuto da Criança e do Adolescente e leis complementares, persistem obstáculos estruturais na atuação dos serviços de saúde, como a fragmentação entre setores, a subnotificação dos casos e a ausência de fluxos bem definidos para o encaminhamento das vítimas^(1,2)^. As estatísticas revelam um aumento expressivo dos casos de abuso, sobretudo sexual, contra meninas de até 13 anos (Figura 1), majoritariamente praticados por familiares, indicando a necessidade urgente de fortalecer a articulação intersetorial e qualificar os profissionais para a detecção precoce e o atendimento humanizado. Portanto, a maioria das vítimas de estupro no Brasil são meninas, não mulheres^(3,4)^.

**Figura 1:** Faixa etária das vítimas de estupro e estupro de vulnerável (%), Brasil, 2023

*Fonte: Fórum Brasileiro de Segurança Pública, 2024.*

A violência infantil é um fenômeno multifacetado, com raízes em determinações históricas, sociais e culturais que se reproduzem no cotidiano das famílias e instituições. Envolve diferentes formas de agressão - física, sexual, psicológica, negligência - e configura violação grave dos direitos da criança e do adolescente, reconhecida internacionalmente como fator de risco para diversos agravos em saúde física e mental^(5)^. Os agressores são, na maioria das vezes, pessoas próximas, como pais e responsáveis^(3)^, o que dificulta o reconhecimento da violência e o rompimento do ciclo de abuso^(2)^. Crianças com deficiência enfrentam riscos ainda maiores, uma vez que a escassez de dados específicos sobre esse grupo dificulta a formulação de intervenções eficazes e a implementação de políticas direcionadas à sua proteção^(6)^.

Segundo dados do 18º Anuário Brasileiro de Segurança Pública (2024), em 2023, foram registrados mais de 56 mil estupros de vulneráveis no Brasil, sendo 88,7% das vítimas do sexo feminino e 61,4% com até 13 anos de idade. Além disso, 64,4% dos agressores faziam parte do convívio familiar da criança. Tais dados reforçam que o lar, idealizado como espaço de segurança e afeto, pode se tornar o principal local de violação e sofrimento para milhares de crianças^(4)^.

As consequências da violência na infância são múltiplas e duradouras, afetando diretamente o desenvolvimento neuropsicomotor, a aprendizagem, o comportamento e a formação de vínculos saudáveis. Crianças expostas à violência têm maior probabilidade de desenvolver transtornos mentais, como depressão e ansiedade, além de apresentar prejuízos escolares, uso precoce de substâncias psicoativas e repetição de padrões de violência na vida adulta^(7,8)^.

A pandemia de COVID-19 trouxe agravantes para o fenômeno da violência contra a criança, ao gerar o fechamento temporário de escolas e serviços assistenciais, dificultando a identificação precoce da violência e ampliando a exposição das crianças a contextos de risco. A literatura mostra que o isolamento social, o estresse parental e a sobrecarga econômica funcionaram como gatilhos para o aumento da violência doméstica durante o período pandêmico. A retração das políticas públicas e a redução de atendimentos presenciais comprometeram ainda mais a resposta intersetorial^(9,10)^.

A produção científica sobre o tema, embora crescente, ainda é insuficiente diante da magnitude do problema. Há carência de estudos avaliativos e investigações com rigor metodológico que permitam mensurar o impacto das ações em saúde sobre os indicadores de violência contra crianças^(2)^. Além disso, os estudos apontam a necessidade de abordagens que considerem a interseccionalidade dos sujeitos atendidos - gênero, raça, deficiência, território - como elementos centrais para a construção de práticas mais equitativas^(6)^.

Embora os serviços de saúde sejam porta de entrada para casos de violência, a resposta institucional ainda é insuficiente diante da complexidade das demandas. Estudos apontam para a baixa resolutividade das equipes, dificuldades em identificar sinais não físicos de violência, ausência de fluxos de encaminhamento definidos e escassa articulação com os demais setores da rede de proteção^(1,11)^. A notificação compulsória, prevista em lei como um instrumento essencial de vigilância e proteção, ainda é pouco utilizada na prática cotidiana por profissionais que, muitas vezes, desconhecem sua finalidade ou temem represálias^(12)^.

O enfrentamento efetivo da violência infantil requer uma atuação articulada entre os setores da saúde, educação, assistência social e justiça, conforme preconizado nas políticas públicas brasileiras e nos compromissos internacionais assumidos pelo país. A Agenda 2030 para o Desenvolvimento Sustentável, da qual o Brasil é signatário, destaca a erradicação de todas as formas de violência contra crianças como um imperativo ético e político para o desenvolvimento justo e sustentável. Em especial, o Objetivo de Desenvolvimento Sustentável (ODS) 16 propõe “promover sociedades pacíficas e inclusivas, proporcionar acesso à justiça para todos e construir instituições eficazes, responsáveis e inclusivas em todos os níveis”^(13)^.

Para que os serviços de saúde possam contribuir com o alcance do ODS 16, é necessário consolidar práticas baseadas em evidências, fortalecer os vínculos comunitários e implementar protocolos institucionais que orientem o acolhimento, a escuta qualificada, a notificação e o encaminhamento dos casos^(12,14)^. A formação em direitos humanos, a abordagem centrada na vítima e o trabalho interprofissional são componentes indispensáveis para uma atuação comprometida com a dignidade e o bem-estar da infância^(13,15)^.

A enfermagem, por sua capilaridade e contato direto com a população, desempenha papel fundamental na identificação precoce da violência, no acolhimento às vítimas e no acompanhamento dos casos. Estudos demonstram que profissionais de enfermagem capacitados conseguem detectar sinais sutis de sofrimento infantil e atuar de forma proativa na mobilização da rede de proteção. No entanto, para que essa atuação se efetive, é imprescindível garantir suporte institucional, tempo adequado de consulta e ambientes seguros para a realização do cuidado^(16,17)^.

Diante do exposto, torna-se evidente que a violência doméstica contra crianças é um problema de alta relevância, cuja abordagem exige respostas integradas baseadas em evidências científicas e orientadas pelos direitos humanos. Os serviços de saúde devem estar preparados não apenas para atender às consequências da violência, mas também para atuar de forma preventiva, educativa e articulada. Este estudo é justificado pela necessidade de sistematizar o conhecimento existente sobre o tema, identificar lacunas na atuação em saúde e propor caminhos para o fortalecimento da proteção integral à infância. Assim, a pesquisa busca responder à seguinte questão: o que a literatura apresenta sobre os limites e as potencialidades dos serviços de saúde para o enfrentamento da violência doméstica contra as crianças no contexto da rede intersetorial?

## OBJETIVOS

Mapear e analisar estudos acerca dos limites e das potencialidades dos serviços de saúde para o enfrentamento da violência doméstica contra crianças no contexto da rede intersetorial à luz das metas do ODS 16, que propõe o fortalecimento de instituições eficazes, inclusivas e responsáveis para a promoção da paz, justiça e proteção infantil.

## MÉTODOS

Trata-se de revisão de escopo seguindo o *Preferred Reporting Items for Systematic Reviews and Meta-Analyses Extension for Scoping Reviews* (Figura 2), cujos objetivos, critérios de inclusão e métodos foram definidos previamente, além do registro do protocolo^(18)^. A revisão foi elaborada utilizando o método recomendado pelo JBI^(19)^, consistindo em revisão exploratória^(20)^. Foram realizados procedimentos de seleção e extração de dados a partir de estudos publicados em bases científicas, seguidos por uma análise comparativa entre os dados empíricos coletados e o modelo proposto. As buscas foram realizadas no período de julho a novembro de 2024, em periódicos indexados nas bases de dados por meio do portal da Coordenação de Aperfeiçoamento de Pessoal de Nível Superior (CAPES) e da Biblioteca Virtual em Saúde (BVS), como a *Scientific Electronic Library Online* (SciELO), portal *National Library of Medicine* (PubMed) e EMBASE, inicialmente como primeira tentativa de busca. Essa estratégia inicial possibilitou a formulação de um método de busca estruturado que foi posteriormente ajustado e adaptado para outras bases de dados, como a Base de Dados de Enfermagem (BDENF), CINAHL, Cochrane, LILACS, Scopus, *Web of Science, Open Grey* e ProQuest.

**Figura 2:** Preferred Reporting Items for Systematic Reviews and Meta-Analyses Extension for Scoping Reviews (PRISMA-ScR), 2024

Estudos em português, inglês e espanhol, publicados a partir de 2000, revisados por pares e alinhados aos ODS 16, envolvendo situações de violência contra crianças no contexto doméstico, enfrentamento da violência doméstica contra crianças pelos serviços de saúde no contexto da rede intersetorial e práticas realizadas por profissionais de saúde vinculadas à rede intersetorial de atendimento especializada e não especializada, foram incluídos. Estudos sobre enfrentamento da violência contra crianças em situação de guerra, conflito armado ou em abrigamento institucional, descrição de projetos, protocolos e programas de enfrentamento à violência contra a criança, além de artigos de revisão, teses, dissertações, livros, diretrizes de organizações, cartas, editoriais, resumos, políticas institucionais e estudos apenas com resumo ou resultados parciais ou que não estavam disponíveis *online*, foram excluídos.

A análise dos achados foi conduzida segundo o referencial metodológico análise de conteúdo de Bardin^(21)^, que se caracteriza por uma abordagem sistemática, embora admita certo grau de subjetividade, empregando a indução e a intuição como estratégias para aprofundar a compreensão dos fenômenos investigados. No contexto da pesquisa qualitativa, Bardin^(21)^ propõe que a análise seja estruturada em três etapas sucessivas: pré-análise; exploração do material; e tratamento dos resultados, que envolve inferências e interpretações.

Nesse processo, a teoria emerge da própria análise, sendo as categorias desenvolvidas progressivamente à medida que os dados são refinados. Conforme Bardin^(21)^, a categorização ocorre por meio da classificação dos elementos com base em critérios de diferenciação, seguida do reagrupamento dos dados de acordo com os temas que emergem da análise, o que caracteriza a definição das categorias *a posteriori* ^(22)^.

Este artigo, derivado de uma dissertação de mestrado, concentra-se especificamente nos elementos relacionados ao primeiro e ao último eixo temático, com o objetivo de fornecer subsídios para a discussão central do estudo.

## RESULTADOS

Foram incluídos 34 documentos (Figura 2), predominantemente de abordagem quantitativa (67,65%). A Tabela 1 apresenta a distribuição dos artigos publicados por país de origem, incluindo o número absoluto de publicações, a porcentagem correspondente e o total geral. O conjunto abrange 34 artigos distribuídos entre 13 países, demonstrando uma amplitude considerável na origem das pesquisas. Os estudos revelaram a predominância de publicações internacionais com concentração de produções nos Estados Unidos da América (EUA) e baixa densidade de investigações latino-americanas.

**Tabela 1:** Resumo dos artigos incluídos

País de origem	N.º	%
Estados Unidos da América	10	29,41
Brasil	5	14,71
Holanda	5	14,71
Reino Unido	4	11,76
Nova Zelândia	2	5,88
Alemanha	1	2,94
Austrália Ocidental	1	2,94
Canadá	1	2,94
Espanha	1	2,94
Japão	1	2,94
Paraguai	1	2,94
Suécia	1	2,94
Vietnã	1	2,94
**Total**	**34**	**100**

Os EUA lideram, com dez estudos, representando 29,41% do total. Brasil e Holanda aparecem empatados em segundo lugar, cada um com cinco estudos, correspondendo a 14,71% cada. O Reino Unido ocupa a terceira posição, com quatro estudos (11,76%), seguido pela Nova Zelândia, com dois estudos (5,88%). Países que apresentam um estudo cada (2,94%) são Alemanha, Austrália Ocidental, Canadá, Espanha, Japão, Paraguai, Suécia e Vietnã.

A Figura 3 apresenta uma produção científica irregular e insuficiente diante da gravidade e abrangência global da violência doméstica contra crianças, especialmente em países com alta prevalência do problema, como o Brasil. Observa-se um número reduzido de publicações, com média de 1,42 publicações anuais (2000-2023), o que limita a construção de evidências contextualmente apropriadas para subsidiar políticas públicas e práticas de cuidado mais efetivas no cenário internacional e brasileiro. Essa lacuna evidencia a necessidade de fomentar pesquisas interdisciplinares e intersetoriais no campo da saúde, com enfoque nos direitos da criança e alinhadas às metas do ODS 16.

**Figura 3:** Artigos selecionados na revisão de escopo segundo o ano de publicação

Os dados de pesquisa estão disponíveis no Quadro 1, que apresenta a síntese dos estudos incluídos, contemplando características como ano de publicação, país de origem, título, autores, objetivos e principais resultados. Adicionalmente, as palavras mais recorrentes encontradas nos estudos incluídos, estão representadas na Figura 4 pela nuvem de palavras.

**Quadro 1:** Resumo dos artigos incluídos na revisão

DOC	ANO PAÍS	TÍTULO	AUTORES	OBJETIVO	PRINCIPAIS RESULTADOS
D01	2005 Estados Unidos da América	*Behaviors of children who are exposed and not exposed to intimate partner violence: an analysis of 330 black, white, and Hispanic children* ^(23)^	Judith M. McFarlane, Janet Y. Groff, Jennifer A. O’Brien & Kathy Watson	Determinar se um programa de tratamento oferecido a mães vítimas de abuso afeta positivamente o comportamento de seus filhos.	Crianças expostas à violência doméstica melhoraram seu comportamento após 24 meses, especialmente entre 18 meses e 5 anos. Avaliações e encaminhamentos foram eficazes, mas adolescentes ainda enfrentaram alta depressão e ansiedade. A maioria superou as normas de referência após 24 meses.
D02	2007 Estados Unidos da América	*What criteria do Child Protective Services investigators use to substantiate exposure to domestic violence?* ^(24)^	Carol Coohey	Determinar se os investigadores dos serviços de proteção à criança aplicam um conjunto reconhecível de critérios para comprovar que agressores e vítimas de agressão expõem seus filhos à violência doméstica.	O estudo utilizou critérios consistentes para a identificação de casos de violência doméstica, priorizou a responsabilização dos agressores e determinou a remoção da criança apenas em situações de risco iminente, com foco nas circunstâncias de maior ameaça à sua segurança.
D03	2008 Brasil	Detecção de maus-tratos contra a criança: oportunidades perdidas em serviços de emergência na cidade do RJ, Brasil^(25)^	Anna Tereza Miranda Soares de Moura, Claudia Leite Moraes, Michael Eduardo Reichenheim	Estimar a magnitude da violência psicológica, violência física e negligência vivenciada por crianças que frequentam estes serviços.	A busca ativa revelou alta subnotificação, exigindo melhor treinamento, organização dos serviços e integração para proteger crianças vítimas de violência.
D04	2008 Nova Zelândia	*Improving detection and quality of assessment of child abuse and partner abuse is achievable with a formal organisational change approach* ^(26)^	Russell Wills, Miranda Ritchie, Mollie Wilson	Melhorar a detecção e a qualidade da avaliação do abuso infantil e do parceiro em um serviço de saúde.	O estudo apontou um aumento significativo nas notificações e na identificação de abuso infantil por serviços de saúde, além de melhorias nos registros clínicos e encaminhamentos. A capacitação de mais de 700 profissionais resultou em maior confiança para triagens e intervenções, mostrando uma sustentabilidade e aumento contínuo nas práticas.
D05	2009 Paraguai	*Child Abuse: the Experience of the Multi-Disciplinary Mental Health Unit of the Niños de Acosta Ñú General Pediatric Hospital* ^(27)^	Lourdes Zelaya de Migliorisi, Lic. Estela González, Lic. Lina Piris de Almirón	Descrever as características epidemiológicas, os motivos mais frequentes de consulta e os diagnósticos associados ao abuso infantil.	A violência intrafamiliar e sintomas depressivos foram os principais motivos de consulta infantil, com 56,5% das vítimas entre 4 e 14 anos. Pediatras precisam de capacitação para uma abordagem multidisciplinar, já que 99% das crianças conheciam o agressor, e negligência foi o tipo mais frequente de maltrato.
D06	2010 Estados Unidos da América	*Children who witness violence: what services do they need to heal?* ^(28)^	Danny Willis, Joellen W. Hawkins, Carole W. Pearce, Jaime Phalen, Meredith Keet, Cristen Singer	Obter informações em grupos focais sobre intervenções que os participantes com experiências pessoais e profissionais com crianças que são testemunhas de violência acreditam que podem ser úteis para as crianças, adolescentes e suas famílias.	As intervenções multissetoriais foram importantes para promover resiliência em crianças expostas à violência, enfatizando a capacitação de profissionais, apoio emocional com programas criativos e coalizões comunitárias colaborativas para enfrentar a violência de forma eficaz.
D07	2010 Reino Unido	*Safeguarding children and public health: midwives’ responsibilities* ^(29)^	Anne Lazenbatt	Comparar e contrastar como as parteiras que trabalham em hospitais ou ambientes comunitários estão atualmente respondendo à coocorrência de abuso doméstico e infantil.	A pesquisa evidenciou a forte relação entre violência doméstica e abuso infantil, destacando a alta incidência de agressões durante a gravidez e os riscos elevados para crianças pequenas. Além disso, identificou falhas no treinamento e na capacidade de relato de parteiras, ressaltando a necessidade urgente de capacitação e abordagens multidisciplinares para uma resposta mais eficaz.
D08	2011 Holanda	*Addressing risk factors for child abuse among high risk pregnant women: design of a randomised controlled trial of the nurse family partnership in Dutch preventive health care* ^(30)^	Jamila Mejdoubi, Silvia van den Heijkant, Elle Struijf, Frank van Leerdam, Remy HiraSing, Alfons Crijnen	Avaliar se o *VoorZorg* é tão eficaz na Holanda quanto nos Estados Unidos da América.	O programa *VoorZorg* mostrou eficácia na prevenção do abuso infantil e violência doméstica ao fornecer suporte contínuo e intervenções estruturadas às mães, abordando fatores de risco por meio de visitas domiciliárias e treinamento em habilidades parentais. Ele melhorou a saúde materna e infantil, criando um ambiente mais seguro e reduzindo a perpetuação de ciclos de violência.
D09	2011 Espanha	*Maternal experiences of childhood abuse and intimate partner violence: Psychopathology and functional impairment in clinical children and adolescents* ^(31)^	Jenniffer K. Miranda, Nuria de la Osa, Roser Granero, Lourdes Ezpeleta	Examinar os efeitos independentes do abuso infantil e da violência por parceiro íntimo das mães na psicopatologia e no comprometimento funcional em crianças; verificar o papel moderador e mediador potencial de fatores individuais e familiares nesses relacionamentos; explorar os efeitos cumulativos potenciais do abuso infantil e da violência por parceiro íntimo maternos nos resultados das crianças.	Crianças expostas à violência doméstica, incluindo abuso materno e punição física, apresentam risco elevado de transtornos psicológicos, como problemas comportamentais e internalização. A exposição cumulativa a múltiplas formas de violência agrava esses impactos, destacando a necessidade de intervenções precoces e apoio às mães para mitigar os efeitos negativos.
D10	2012 Alemanha	*Key role in the prevention of child neglect and abuse in Germany: continuous care by qualified family midwives* ^(32)^	Gertrud M. Ayerle, Katja Makowsky, Beate A. Schucking	Obter um conhecimento aprofundado das famílias vulneráveis psicossociais e relacionadas à saúde e do “portfólio” de cuidados que as parteiras familiares fornecem.	A violência doméstica é um fator de risco significativo enfrentado por muitas famílias atendidas, com 21,5% relatando experiências de violência e padrões de vulnerabilidade complexos. A colaboração com serviços de bem-estar juvenil e atividades educacionais contínuas foram essenciais para abordar esses desafios e melhorar o cuidado materno-infantil.
D11	2013 Estados Unidos da América	*A brief intervention affects parents’ attitudes toward using less physical punishment* ^(33)^	Antwon Chavis, Julia Hudnut-Beumler, Margaret W. Webb, Jill A. Neely, Len Bickman, Mary S. Dietrich, Seth J. Scholer	Determinar se uma intervenção breve, integrada à visita de atenção primária, pode afetar as atitudes dos pais em relação ao uso de menos punição física.	Uma breve intervenção multimídia reduziu as atitudes dos pais favoráveis ao uso de punição física e aumentou o uso de estratégias de disciplina não violentas.
D12	2013 Holanda	*A new protocol for screening adults presenting with their own medical problems at the Emergency Department to identify children at high risk for maltreatment* ^(34)^	Hester M. Diderich, Minne Fekkes, Paul H. Verkerk, Fieke D. Pannebakker, Mariska Klein Velderman, Peggy J.G. Sorensen, Paul Baeten, Anne Marie Oudesluys-Murphy	Avaliar se um protocolo de triagem de adultos que se apresentam para atendimento no Departamento de Emergência pode identificar crianças com alto risco de tratamento formal.	A implementação do protocolo de Haia aumentou significativamente a detecção de casos de maus-tratos infantis, com alta taxa de confirmação (91%), e revelou que a maioria dos casos era desconhecida das autoridades. A detecção incluiu principalmente crianças expostas à violência doméstica, comprovando a eficácia do protocolo em identificar novos casos de abuso infantil.
D13	2013 Estados Unidos da América	*Building healthy children: evidence-based home visitation integrated with pediatric medical homes* ^(35)^	Heather A Paradis, Mardy Sandler, Jody Todd Manly, Laurie Valentine	Evitar maus-tratos infantis, melhorar a saúde dos pais e dos filhos, e aprimorar o funcionamento familiar.	O programa *Building Healthy Children* reduziu a exposição infantil à violência doméstica e a necessidade de relatórios ao *Child Protective Services*, garantindo alta retenção de participantes (85%) e prevenindo novos casos de abuso, além de promover cuidados preventivos para um desenvolvimento saudável.
D14	2013 Brasil	As possibilidades de enfrentamento da violência infantil na consulta de enfermagem sistematizada^(36)^	Maíra Rosa Apostólico, Paula Hino, Emiko Yoshikawa Egry	Identificar os limites e potencialidades da Classificação Internacional das Práticas de Enfermagem em Saúde Coletiva^®^ na consulta de enfermagem com crianças vítimas de violência doméstica.	A nomenclatura Classificação Internacional das Práticas de Enfermagem em Saúde Coletiva^®^ apresentou limitações no reconhecimento da violência, especialmente em casos de negligência e violência infantil, dificultando a notificação e a continuidade da assistência. A falta de precisão nos diagnósticos evidenciou a necessidade de atributos adicionais e maior integração com protocolos para garantir um atendimento mais eficaz.
D15	2014 Holanda	*Facilitators and barriers to the successful implementation of a protocol to detect child abuse based on parental characteristics* ^(37)^	Hester M. Diderich, Mark Dechesne, Minne Fekkes, Paul H. Verkerk, Fieke D. Pannebakker, Mariska Klein Velderman, Peggy J.G. Sorensen, Simone E. Buitendijk, Anne Marie Oudesluys-Murphy	Explorar se as diretrizes do Protocolo de Haia podem ser implementadas com sucesso e identificar facilitadores ou barreiras críticas à implementação.	A implementação do Protocolo de Haia aumentou significativamente as referências de casos de abuso infantil ao *Reporting Center for Child Abuse and Neglect*, porém a baixa taxa de confirmação em algumas regiões e a falta de treinamento dos profissionais comprometeram a eficácia. A presença de um *coach* de implementação e a capacitação contínua dos profissionais foram essenciais para melhorar a detecção e resposta ao abuso infantil em contextos variados.
D16	2014 Estados Unidos da América	*Health, emergency department use, and early identification of young children exposed to trauma* ^(38)^	Yvonne Humenay Roberts, Cindy Y. Huang, Cindy A. Crusto, Joy S. Kaufman	Descrever a prevalência de sintomas de saúde física e problemas relacionados à saúde em crianças pequenas afetadas por traumas e prever se crianças que sofrem traumas têm ou não maior probabilidade de serem afetadas por problemas relacionados à saúde.	A alta prevalência de trauma em crianças expostas à violência doméstica levou a um aumento no uso dos serviços de saúde, evidenciando a necessidade de triagem e intervenção adequadas nos departamentos de emergência. Treinamento especializado para profissionais de saúde foi essencial para melhorar a identificação precoce e o cuidado de crianças afetadas por trauma.
D17	2014 Reino Unido	*Keeping the focus on children: the challenges of safeguarding children affected by domestic abuse* ^(39)^	Sue Peckover, Fiona Trotter	Examinar os desafios enfrentados pelos profissionais na proteção de crianças afetadas por violência doméstica, particularmente aqueles que trabalham em serviços de apoio universais e adicionais (DH 2009).	Profissionais reconhecem o impacto da violência doméstica em crianças, mas enfrentam dificuldades em agir devido à falta de serviços especializados, treinamento adequado e foco nas necessidades infantis. Além disso, a delegação da responsabilidade a outras agências gera lacunas no atendimento.
D18	2014 Holanda	*Support and monitoring of families after child abuse detection based on parental characteristics at the Emergency Department* ^(40)^	H M Diderich, F D Pannebakker, M Dechesne, S E Buitendijk, A M Oudesluys-Murphy	Investigar o que aconteceu com as famílias três meses após detecção de abuso.	O uso do protocolo de Haia no atendimento emergencial foi eficaz para identificar casos de abuso infantil, com 91% dos casos encaminhados sendo confirmados. No entanto, houve limitações no monitoramento contínuo e na colaboração entre serviços, o que leva a reencaminhamentos frequentes e indica a necessidade de um sistema centralizado de acompanhamento para prevenir recorrências.
D19	2015 Estados Unidos da América	*Child maltreatment and risk patterns among participants in a child abuse prevention program* ^(41)^	Jennifer Y. Duffy, Marcia Hughes, Andrea G. Asnes, John M. Leventhal	Examinar a relação entre os fatores de risco parentais e o *status* de comprovação e o número de relatórios do Serviço de Proteção das Crianças em famílias em um programa de prevenção em todo o estado.	As famílias de alto risco social atendidas por um programa de prevenção ao abuso infantil e denunciadas ao Serviço de Proteção das Crianças apresentaram alto risco parental. O histórico de violência e criminalidade dos pais, a presença de múltiplos cuidadores e pelo menos dois dos seis fatores de risco foram associados à ocorrência ou reincidência de maus-tratos.
D20	2015 Holanda	*Detecting child abuse based on parental characteristics: Does The Hague Protocol cause parents to avoid the Emergency Department?* ^(42)^	Hester M. Diderich RN, Minne Fekkes, Mark Dechesne, Simone E. Buitendijk MD, Anne Marie Oudesluys-Murphy	Investigar se a implementação do Protocolo de Haia pode diminuir o atendimento médico de emergência.	O estudo revelou que, após a implementação do Protocolo de Haia, o número de pacientes atendidos no departamento de emergência seguiu elevado e até aumentou, indicando que o protocolo não afasta os pacientes.
D21	2015 Estados Unidos da América	*Intimate Partner Violence Programs in a Children’s Hospital: Comprehensive Assessment Utilizing a Delphi Instrument* ^(43)^	Kimberly A. Randell, Sarah E. Evans, Donna O’Malley, M. Denise Dowd	Realizar uma avaliação inicial das práticas de violência entre parceiros íntimos em um sistema hospitalar pediátrico.	O estudo identificou limitações nas práticas de triagem e resposta à violência doméstica no hospital, com baixa pontuação em práticas de intervenção, falta de treinamento e protocolos padronizados, além de barreiras institucionais e individuais que dificultam a implementação de uma abordagem coordenada e eficaz.
D22	2015 Brasil	Violência infantil: uma análise das notificações compulsórias, Brasil 2011^(44)^	Susana Maria Moreira Rates, Elza Machado de Melo, Márcio Dênis Medeiros Mascarenhas, Deborah Carvalho Malta	Analisar notificações de violências contra crianças entre 0 e 9 anos registradas pelos serviços públicos de saúde no Brasil.	O estudo revelou que a violência doméstica contra crianças no Brasil ocorre majoritariamente no ambiente familiar, com alta prevalência de negligência e repetição dos abusos, destacando a necessidade de ações intersetoriais, capacitação profissional e intervenções direcionadas a pais e cuidadores para efetiva proteção infantil.
D23	2016 Nova Zelândia	*Are mental health services getting better at responding to abuse, assault and neglect?* ^(45)^	Read J, Sampson M, Critchley C	Determinar se as respostas da equipe às denúncias de abuso melhoraram desde a introdução de uma política de trauma e programa de treinamento.	O estudo revelou que, apesar da maior inclusão do abuso nos planos de tratamento após políticas de trauma, ainda existem falhas significativas, como baixa taxa de encaminhamentos, respostas inadequadas à negligência e falta de vínculo entre abuso infantil e problemas de saúde mental, destacando a necessidade de protocolos mais eficazes.
D24	2016 Brasil	Impacto da violência por parceiro íntimo no uso de cuidados primários de saúde para crianças: evidências do RJ, Brasil^(46)^	Claudia Leite de Moraes, Aline Gaudard e Silva de Oliveira, Michael Eduardo Reichenheim	Avaliar se a violência física grave entre parceiros íntimos após o parto afeta o número de consultas pediátricas em unidades de Atenção Primária à Saúde durante esse período.	Mães que sofreram violência severa do parceiro após o parto frequentam menos serviços pediátricos, prejudicando o desenvolvimento infantil. O impacto é maior em crianças com problemas de saúde. A detecção precoce da violência por profissionais de saúde é crucial para proteção e cuidado adequado.
D25	2017 Reino Unido	*Training on domestic violence and child safeguarding in general practice: a mixed method evaluation of a pilot intervention* ^(47)^	Natalia V. Lewis, Cath Larkins, Nicky Stanley, Eszter Szilassy, William Turner, Jessica Drinkwater, Gene S. Feder	Testar e avaliar a viabilidade, aceitabilidade e direção da mudança em medidas de resultados de curto prazo de um programa piloto de treinamento baseado em evidências sobre violência doméstica e proteção infantil para equipes de clínica geral.	O treinamento *Researching Education to Strengthen Primary care on Domestic Violence and Safeguarding* melhorou significativamente o conhecimento e a confiança dos clínicos, mas teve impacto limitado na mudança de comportamento clínico e enfrentou desafios na colaboração intersetorial, apontando a necessidade de refinamento e maior apoio para implementação eficaz.
D26	2018 Japão	*Cumulative risk effect of household dysfunction for child maltreatment after intensive intervention of the child protection system in Japan: a longitudinal analysis* ^(48)^	Hirotsuna Ohashi, Ichiro Wada, Yui Yamaoka, Ryoko Nakajima-Yamaguchi, Yasukazu Ogai, Nobuaki Morita	Investigar como a disfunção familiar e múltiplos tipos de maus-tratos preveem a reincidência de maus-tratos infantis após intervenções no sistema de proteção infantil no Japão.	Disfunções familiares como violência e abuso de substâncias elevam o risco de recorrência de maus-tratos infantis, mesmo após intervenções. Um modelo de risco cumulativo indica que múltiplas disfunções aumentam essa vulnerabilidade. Isso reforça a necessidade de serviços pós-custódia e cooperação intersetorial para proteção eficaz.
D27	2018 Brasil	Proteção de crianças e adolescentes vítimas de violência: a visão dos profissionais de um serviço especializado^(49)^	Priscila Arruda da Silva, Valéria Lerch Lunardi, Rodrigo Dalke Meucci, Simone Algeri	Conhecer os obstáculos enfrentados pelos profissionais para trabalhar em rede e os desafios da sua atuação no Centro de Referência Especializado de Assistência Social de um município do extremo sul do Brasil.	A fragmentação da rede de atendimento, a burocracia, a escassez de recursos e a comunicação ineficaz comprometem a proteção de crianças vítimas de violência. Profissionais sobrecarregados, sem treinamento adequado e com suporte insuficiente enfrentam dificuldades que reduzem a efetividade dos serviços.
D28	2019 Austrália Ocidental	*Hospitalisations for maternal assault are associated with increased risk of child protection involvement* ^(50)^	Carol Orr, Colleen Fisher, Scott Sims, David Preen, Rebecca Glauert, Melissa O’Donnell	Examinar o risco de alegações de maus-tratos em crianças cujas mães foram hospitalizadas devido a uma agressão.	Os filhos de mães hospitalizadas por agressão, especialmente durante a gestação, apresentaram risco significativamente elevado de alegações de maus-tratos. Destaca a urgência de intervenções precoces, colaboração intersetorial e maior conscientização dos profissionais para apoiar famílias vulneráveis.
D29	2019 Suécia	*Reduced psychiatric symptoms at 6 and 12 months’ follow-up of psychotherapeutic and psychoeducative group interventions for children exposed to intimate partner violence* ^(51)^	Karin Perneboa, Mats Fridell, Kjerstin Almqvist	Investigar os resultados de longo prazo de duas intervenções de grupo estabelecidas para crianças expostas à violência por parceiro íntimo e seus pais não agressores.	O estudo destacou melhorias sustentadas nos sintomas de estresse e comportamento infantil após intervenções contra violência doméstica, com maior benefício para crianças com sintomas traumáticos severos. A continuidade de melhorias foi influenciada pela saúde psicológica materna, e houve pouca renovação de exposição à violência.
D30	2020 Vietnã	*Clinician Response to Child Abuse Presentations in the Vietnamese Hospital Emergency Setting* ^(52)^	Tara Flemington, Cathrine Fowler, Quang Nhat Tran, Jennifer Fraser	Explorar o conhecimento, a prática e a experiência de clínicos quando confrontados com apresentações de abuso infantil em um ambiente de emergência pediátrica.	O estudo evidenciou que profissionais de saúde no Vietnã enfrentam barreiras como falta de treinamento, protocolos inadequados e dificuldades na documentação de abuso infantil. Apesar disso, desenvolveram métodos inovadores de proteção e enfatizam a necessidade de treinamentos e diretrizes claras para aprimorar a resposta aos casos.
D31	2021 Estados Unidos da América	*Evaluating child maltreatment and family violence risk during the COVID-19 Pandemic: Using a telehealth home visiting program as a conduit to families* ^(53)^	Lindsey Rose Bullinger, Stevan Marcus, Katherine Reuben, Daniel Whitaker, Shannon Self-Brown	Examinar como as famílias com crianças pequenas são afetadas por dificuldades da pandemia de COVID-19 e avaliar as mudanças nas percepções dos profissionais sobre o risco de maus-tratos devido a essas circunstâncias incomuns.	O estudo mostrou que a pandemia de COVID-19 aumentou significativamente o risco de maus-tratos infantis, impulsionado por estresse parental, dificuldades financeiras e falta de distanciamento social. A eficácia do programa *SafeCare* foi destacada como uma intervenção importante, mas insuficiente diante das barreiras enfrentadas pelas famílias vulneráveis.
D32	2021 Reino Unido	*‘It felt like there was always someone there for us’: Supporting children affected by domestic violence and abuse who are identified by general practice* ^(54)^	Jessica Roy, Emma Williamson, Katherine Pitt, Nicky Stanley, Mei-See Man, Gene Feder, Eszter Szilassy	Verificar se o treinamento e apoio de advocacia IRIS+ (*Enhanced Identification and Referral to Improve Safety*) melhorou a forma como os clínicos respondem a crianças e jovens afetados por violência doméstica e abuso.	O estudo revelou falhas significativas na identificação e encaminhamento de crianças expostas à violência doméstica, com barreiras como falta de clareza dos profissionais e medo das mães. Apesar disso, o suporte especializado trouxe benefícios importantes, melhorando o bem-estar infantil e a comunicação familiar.
D33	2021 Canadá	*Screening for intimate partner violence in the early postpartum period: Maternal and child health and social outcomes from birth to 5-years post-delivery* ^(55)^	Tamara L. Taillieu, Douglas A. Brownridge, Marni Brownell	Examinar a saúde materna e infantil, bem como os resultados sociais do nascimento até 5 anos após o parto, associados a uma triagem de violência por parceiro íntimo materna positiva (*vs*. negativa) na época do parto.	Crianças expostas à violência doméstica enfrentam maiores riscos de transtorno do déficit de atenção com hiperatividade, infecções respiratórias, hospitalizações por lesões e desenvolvimento comprometido, enquanto as mães apresentam problemas de saúde mental e física. Esses achados destacam a necessidade de intervenções integradas entre saúde e proteção social para apoiar as famílias afetadas.
D34	2023 Estados Unidos da América	*Evaluation of Children after Caregiver Intimate Partner Violence: A Qualitative Study of Barriers, Facilitators, and Traumaand Violence-Informed Care* ^(56)^	Gunjan Tiyyagura, Nicole Clayton, Paula Schaeffer, Marcie Gawel, John M. Leventhal, Kristen Hammel, Karen Jubanyik, Destanee Crawley, Ashley Frechette, Daniel M. Lindberg, Tami Sullivan, Andrea Asnes	Identificar barreiras e facilitadores na avaliação de crianças expostas à violência por parceiro íntimo e desenvolver uma estratégia para otimizar a avaliação.	A avaliação médica de crianças expostas à violência doméstica permitiu identificar abusos físicos e conectar cuidadores a recursos de apoio, apesar de enfrentar barreiras como a falta de dados e resistência dos pais. Melhor colaboração entre serviços e uma abordagem informada por trauma podem aprimorar o atendimento e o suporte para essas famílias.

**Figura 4:** Nuvem de palavras dos estudos selecionados

Seis categorias empíricas foram identificadas e relacionadas às metas do ODS 16: 1. Prevenção e suporte para violência doméstica e abuso infantil; 2. Intervenções baseadas em treinamento profissional e multidisciplinaridade; 3. Desafios da rede e barreiras profissionais; 4. Riscos e consequências da violência; 5. Importância de abordagens comunitárias e intersetoriais; e 6. Acompanhamento e documentação insuficientes (Quadro 2).

**Quadro 2:** Análise das categorias empíricas à luz das metas do Objetivo de Desenvolvimento Sustentável 16

CATEGORIAS	META 16.1	META 16.2	META 16.3
1. Prevenção e suporte para violência doméstica e abuso infantil	Focar nas intervenções preventivas e programas de suporte que visam reduzir a violência doméstica e o abuso infantil, alinhando-se com a meta de redução da violência.	Focar na eliminação do abuso contra as crianças.	---
2. Intervenções baseadas em treinamento profissional e multidisciplinaridade	---	O treinamento profissional e a abordagem multidisciplinar contribuem para identificar e combater o abuso infantil.	Promover o acesso à justiça por meio de serviços qualificados.
3. Desafios da rede e barreiras profissionais	Superar barreiras requer capacitação, governança intersetorial e sistemas integrados para garantir respostas eficazes e humanizadas à violência.	O fortalecimento de protocolos, a integração dos serviços e o investimento em treinamento profissional são ações essenciais para aprimorar a proteção infantil.	Abordar obstáculos ao acesso à justiça e atendimento, relacionando-se diretamente com a promoção do Estado de Direito e acesso igualitário à justiça.
4. Riscos e consequências da violência	Analisar os impactos e riscos da violência, contribuindo para o entendimento necessário para reduzir os danos associados a essa violência.	Combater o abuso infantil.	---
5. Importância de abordagens comunitárias e intersetoriais	As abordagens comunitárias e intersetoriais são fundamentais para reduzir a violência.	As iniciativas intersetoriais e comunitárias mencionadas são fundamentais para criar redes de proteção eficazes, fortalecendo a resiliência comunitária e a prevenção de todas as formas de abuso infantil.	Garantir acesso à justiça.
6.Acompanhamento e documentação insuficientes	---	A documentação adequada é crucial para garantir que o abuso infantil seja notificado e tratado.	A documentação inadequada e a falta de relatórios limitam a resposta institucional a casos de abuso. Garantir processos legais eficazes e a proteção de vítimas, contribuindo para o fortalecimento do Estado de Direito.

Essas categorias demonstram que os limites enfrentados pelas equipes não se restringem ao nível individual, mas envolvem barreiras sistêmicas e estruturais (D01, D02, D03, D04, D07), como descontinuidade de políticas, ausência de protocolos intersetoriais e fragilidade das redes de apoio (D10, D12, D14, D15, D17, D19, D20, D22, D23, D24, D26, D27, D33, D34) (Quadro 1).

As categorias apontaram que as potencialidades, como as triagens rotineiras (D03, D04, D12, D15, D18, D21, D24, D33), visitas domiciliárias (D08, D10, D11, D13, D19, D31) e programas educacionais (D06, D07, D08, D09, D14, D17, D27, D29, D31, D33), são métodos eficazes para detectar precocemente casos de abuso infantil e violência doméstica, porém demandam profissionais altamente capacitados e recursos significativos (D10, D17, D19, D31, D34).

## DISCUSSÃO

Os estudos revelaram um padrão recorrente de limitações, como a desarticulação entre setores da rede de proteção, a subnotificação dos casos, a escassez de protocolos padronizados e a insuficiência de capacitação contínua dos profissionais de saúde^(12)^. Esses entraves foram recorrentes nas categorias empíricas relacionadas à desarticulação intersetorial, à subnotificação dos casos e à escassez de ações sistematizadas para o acompanhamento das vítimas^(17,23)^.

A desarticulação entre os setores da rede de proteção compromete o fluxo de encaminhamento e o monitoramento dos casos, refletindo um modelo de cuidado fragmentado e pouco resolutivo^(23)^. A escassez de profissionais capacitados, somada à limitação de recursos institucionais, contribui para a invisibilidade das situações de violência e para a revitimização das crianças nos próprios serviços que deveriam acolhê-las^(17)^. Outro aspecto crítico identificado foi a exclusão da voz infantil na formulação das políticas públicas, o que mantém um ciclo de silenciamento institucional das vítimas^(10)^.

A pandemia de COVID-19 agravou o cenário de vulnerabilidade, ao limitar o acesso das crianças aos serviços de proteção e aumentar o tempo de exposição ao agressor. O isolamento social, o estresse parental e a retração das políticas públicas atuaram como fatores de risco adicionais durante o período pandêmico^(6,23)^. Crianças de minorias raciais e étnicas foram desproporcionalmente afetadas, embora a ausência de dados desagregados comprometa análises mais precisas sobre o impacto das desigualdades estruturais^(6)^.

Apesar das limitações, algumas iniciativas se destacam como potencialidades dos serviços de saúde. Os autores identificaram experiências promissoras, como as visitas domiciliares realizadas por profissionais de enfermagem, ações educativas em escolas, atendimento humanizado em serviços de urgência e programas intersetoriais de capacitação, que contribuíram para o fortalecimento da rede de proteção. Tais práticas, no entanto, ainda são pontuais e carecem de institucionalização, financiamento adequado e monitoramento sistemático de resultados^(18)^.

A utilização da Classificação Internacional das Práticas de Enfermagem em Saúde Coletiva^®^ tem contribuído para qualificar a identificação de situações de violência, por meio da sistematização da escuta clínica e da articulação com os serviços de assistência social. Essa ferramenta fortalece a vigilância ativa, o registro adequado dos casos e o planejamento de intervenções mais eficazes^(24)^.

Programas de visitas domiciliares realizados por enfermeiros especializados, como o *VoorZorg*, demonstraram impacto positivo na prevenção da violência, ao promover o fortalecimento dos vínculos familiares e o acompanhamento longitudinal das famílias em situação de risco^(25)^. A parentalidade positiva emergiu como um fator protetivo relevante, especialmente em contextos de alta vulnerabilidade social^(8)^.

Do ponto de vista terapêutico, estratégias como a arteterapia têm se mostrado eficazes na abordagem do trauma infantil, permitindo a expressão emocional da criança por meio de linguagens simbólicas, o que favorece a reconfiguração de vivências violentas e a reconstrução da autoestima. Essas intervenções, embora pontuais, demonstram potencial para serem incorporadas às práticas de cuidado multiprofissional^(24)^.

Além disso, a baixa produção científica sobre o tema, com distribuição geográfica desigual e predominância de estudos com nível metodológico modesto, indica uma lacuna relevante no campo da pesquisa em saúde coletiva e enfermagem. A média de apenas 1,42 publicações anuais ao longo de 24 anos, conforme revelado pelos dados desta revisão, é incompatível com a gravidade e a magnitude do problema, especialmente em países como o Brasil, que figuram entre os mais afetados pela violência contra a criança. Essa escassez reforça a necessidade de investimentos em estudos analíticos, avaliativos e com rigor metodológico capazes de subsidiar políticas públicas efetivas^(26)^.

Os achados dialogam diretamente com as metas do ODS 16, que propõe o fortalecimento de instituições inclusivas, eficazes e responsáveis. A distância entre os compromissos assumidos pelo Brasil e a prática institucional indica a necessidade urgente de políticas públicas estruturadas, com alocação de recursos, formação qualificada e governança colaborativa^(13)^.

### Limitações do estudo

O estudo apresentou algumas limitações, como o recorte temporal adotado, que incluiu estudos publicados a partir do ano 2000. Essa escolha foi fundamentada na vigência do Estatuto da Criança e do Adolescente no Brasil e na adoção, em âmbito internacional, dos ODS da Organização das Nações Unidas, os quais intensificaram a visibilidade das pautas de proteção da infância. Outra limitação foi a exclusão de estudos que não distinguiam claramente a população infantil da adolescente, visando ao foco exclusivo nas crianças, respeitando os recortes etários definidos na literatura e na política de proteção. Nosso entendimento é que existem diferenças relativamente significativas em termos de cuidado e de formas de enfrentamento da violência doméstica entre essas duas populações.

### Contribuições para as áreas da enfermagem, saúde coletiva ou políticas públicas

O estudo detectou lacunas do conhecimento relativamente ao tema e subtemas abordados, especialmente de estudos intensivos, analíticos e atuais sobre as políticas e práticas do enfrentamento em rede intersetorial da violência doméstica contra as crianças.

## CONSIDERAÇÕES FINAIS

A violência doméstica contra crianças impõe desafios urgentes à saúde coletiva, exigindo respostas mais qualificadas e intersetoriais. Este estudo reforça a importância de reposicionar os serviços de saúde como agentes ativos na proteção infantil, não apenas como instâncias de cuidado clínico, mas como estruturas estratégicas de vigilância, escuta e encaminhamento.

Diante da magnitude do problema, torna-se imperativo que as ações de saúde estejam alinhadas aos compromissos éticos e internacionais, como os estabelecidos na Agenda 2030, especialmente o ODS 16, que demanda instituições eficazes, justas e inclusivas. O reconhecimento da criança como sujeito de direitos deve orientar a formulação de políticas públicas e a organização do cuidado, garantindo respostas integradas e baseadas em evidências.

## Data Availability

Os dados de pesquisa estão disponíveis em repositório: https://doi.org/10.5281/zenodo.16423368.
